# The use of complementary and alternative medicine after the completion of hospital treatment for colorectal cancer: findings from a questionnaire study in Denmark

**DOI:** 10.1186/1472-6882-14-388

**Published:** 2014-10-10

**Authors:** Nina Nissen, Anita Lunde, Christina Gundgaard Pedersen, Helle Johannessen

**Affiliations:** Institute of Public Health, University of Southern Denmark, J.B.Winsløws Vej 9B, 5000 Odense C, Denmark; VIA University College, Sundhedsfaglig højskole, Chr. M. Østergaards Vej 4, 8700 Horsens, Denmark; Aarhus University Hospital & Department of Psychology, Aarhus University, Bartholins Allé 9, 8000 Aarhus C, Denmark

## Abstract

**Background:**

Little is known about the use of complementary and alternative medicine (CAM) for colorectal cancer, despite the high incidence of colorectal cancer and the frequency of CAM use for cancer-related symptoms. This is the first Danish study to examine the use of CAM by individuals who completed hospital treatment for colorectal cancer.

**Methods:**

In 2011–12, a pragmatic trial on energy healing as rehabilitation after colorectal cancer was conducted in Denmark with participants who had completed cancer-related hospital treatment within the past 18 months prior to study inclusion. As part of the trial, participants (n = 247) completed a questionnaire on the use, motivations, pathways and perceived benefits of CAM. Socio-demographic information was obtained via the Danish National Patient Registry and self-report. Descriptive statistics were generated, using SPSS, version 18, and logistic regression analysis was carried out.

**Results:**

Of 247 individuals, 49.4% used some form of CAM in the past month. Nearly half of the CAM users (49.2%) used natural medicines and/or dietary supplements only; 32% consulted an alternative therapist; 18.9% used both. Those who consulted alternative therapists were most commonly women (OR: 3.36; p = .002; CI: 1.54-7.33) with high educational levels (OR: 2.77; p = 0.010; CI: 1.28-6.01); more women than men used natural medicines and/or dietary supplements (OR: 1.83; p = .047; CI: 1.01-3.30) independent of educational levels. A majority commenced CAM on their own initiative; CAM was predominantly used to achieve better physical wellbeing. Beneficial effects were reported particularly in relation to physical health; few harmful effects were reported. Of those using CAM, 51.5% did not disclose its use to their physician; 8.5% of participants reported to have been asked by their physician about CAM use.

**Conclusion:**

The use of CAM following completion of hospital treatment for colorectal cancer seems widespread in Denmark. The identified extensive CAM use suggests a need for more reliable and diverse information about CAM for both patients and biomedical providers, and improved communication about its use in the clinical context.

**Electronic supplementary material:**

The online version of this article (doi:10.1186/1472-6882-14-388) contains supplementary material, which is available to authorized users.

## Background

In 2012, colorectal cancer was estimated to be the second most common form of cancer diagnosed in Europe and the second most common cause of death from cancer [1]. In the same year, Denmark was estimated to have the highest age-standardised rate of colorectal cancer incidence in Northern Europe, with the highest mortality rate in women and the third highest mortality rate in men [1]. People with colorectal cancer experience poor quality of life following surgery and treatment, and continue to experience a wide range of side effects after treatment [2–4]. This may make daily activities difficult [5, 6].

Research into patterns of the use of complementary and alternative medicine (CAM) consistently shows that the majority of CAM users in European countries, including Denmark, take recourse to CAM in conjunction with biomedical treatments and for a variety of reasons [7–10]. Main reasons for CAM use focus on symptom relief and the promotion of well-being and quality of life in chronic health conditions and life-threatening illness such as cancer [7–10]. As some CAM use may have clinical implications, including clinically significant drug interactions and potentially adverse interactions with anti-cancer agents [11, 12], knowledge about the use of CAM in cancer and individual cancers is highly relevant.

Despite the frequency of colorectal cancer, little seems to be known about the use of CAM by this group of patients. According to Molassiotis et al’s [10] cross-sectional survey in various European countries (not including Denmark) 32% of people diagnosed with colorectal cancer use some form of CAM post-diagnosis. A colorectal cancer diagnosis is noted to lead to a dramatic increase in CAM use, particularly the use of herbs, medicinal teas and spiritual practices [10]. A population-based study in Alberta (Canada) reports that 49% of colorectal cancer patients used some form of CAM and 69% did so after conventional care [13]; a study based on the colon disease registry at a New York City hospital [14] reports that 75% of colorectal cancer survivors used some form of CAM. CAM use by individuals with colorectal cancer and/or post-completion of cancer treatment, like cancer patients generally, aims to improve general health and wellbeing [10, 15].

In 2011–12, a pragmatic trial on energy healing as rehabilitation after completed hospital treatment for colorectal cancer was conducted in Denmark in order to test guidelines for effectiveness studies measuring personalized treatment goals [16–20]. As part of this trial, an eight-item questionnaire examined participants’ use of CAM in the month prior to inclusion in the trial, with the aim to explore respondents’ prevalence of CAM use, CAM users’ motivations, pathways and perceived benefits and harm resulting from the use of CAM, and communication with physicians about CAM (see Additional file 1). This article reports on this questionnaire, which is the first study in Denmark to have examined the use of CAM by people who have completed hospital treatment for colorectal cancer. The reported CAM use in the past month allows for a snapshot of respondents’ use of CAM, although its use may be unrelated to cancer rehabilitation. To contextualize the identified prevalence of CAM use in colorectal cancer which affects men and women almost equally, findings are compared to CAM use by Danish cancer patients in general, and in relation to breast and prostate cancer, as examples of gender-specific cancers.

## Methods

An eight-item questionnaire about the use of CAM and natural medicines/dietary supplements during the past month constituted part of a larger baseline questionnaire, which was completed by all participants (n = 247) in the pragmatic trial mentioned above. Eligible trial participants were identified from the Danish National Patient Registry and invited to take part in the trial. Eligibility criteria included: 1) primary diagnosis of colorectal cancer, defined as C18- C20 (ICD10); 2) completed treatment with surgery or surgery and chemotherapy and/or radiotherapy in the Southern or Central Region of Denmark between 1 March 2010, and 1 August 2011, and no evidence of current cancer; and 3) aged ≤ 80 at inclusion. Patients were excluded if they: 1) were unable to comply with the data collection protocol, 2) had poor understanding of the Danish language, or 3) were receiving palliative care or had a recurrence of cancer prior to inclusion. Thus, all respondents had completed hospital treatment for colorectal cancer, were considered to be free of cancer disease, and were at different stages of cancer rehabilitation.

The eight-item questionnaire comprised multiple-choice and categorical response questions, including some open response options. The topics examined were: use of listed alternative treatments in the past month; use of natural medicines/dietary supplements in the past month; pathways to the use of alternative treatment and/or natural medicines/dietary supplements; reasons for use and non-use of CAM; communication with physicians about CAM; and perceived beneficial and/or harmful effects resulting from CAM use. To enhance comparability across different studies, the questions were based on a previous questionnaire about CAM use by cancer patients in 14 European countries [21], adapted to the Danish context and piloted at the oncology department at Odense University Hospital, Denmark. The list of complementary and alternative medicine used by Molassiotis et al. [21] was replaced with a list of treatments commonly used in Denmark (see Table 1) [22]. In addition, a single item question measured respondents’ attitude towards CAM, using a 5-point Likert scale ranging from very negative to very positive; the item was developed for the present study instead of using lengthier validated questionnaires that measure attitude.

**Table 1:** **List of alternative treatments and natural medicines and/or dietary supplements**

Alternative treatments	Natural medicines and/or dietary supplements
Massage, osteopathy and other manipulative therapies	For example:
Reflexology	Pills (such as homeopathic pills)
Acupuncture	Tablets or capsules (such as herbal, vitamins/minerals or food supplements)
Healing (laying-on-of-hands or similar)	Medicinal teas
Cranio-sacral therapy	Extracts (such as herbal or homeopathic)
Homeopathy	
Dietary/nutritional therapy	
Kinesiology	
Hypnosis	
Mindfulness/meditation	
Other	

In Denmark, most forms of CAM are provided outside the public healthcare system. In line with Danish regulations and conventions, the questionnaire used the term ‘alternative treatment’ to refer to the field of CAM in general and to individual treatments or therapies; the term ‘natural medicines and/or dietary supplements’ referred to the use of medicinal/nutritional products specifically (see Table 1) [23]. In this article, the term CAM is used when all forms of uses are referred to collectively; the term CAM is also used when referring to international literature, adopting the referenced author/s’ use of the term.

### Ethics

The study adhered to the ethical requirements of the Helsinki Declaration. Participants received both written and oral information about the study, and were informed that they were free to withdraw any time during the study period. The study was presented to the regional Committee of Research Ethics in Southern Denmark; the Committee decided that it did not require their approval. The study was approved by the Danish Data Protection Agency.

### Analysis

Completed questionnaires were digitalised (Teleform software) and the data analysed by AL and CGP using SPSS, version 18. Descriptive statistics were generated and unadjusted associations with use of alternative treatments and natural medicines/dietary supplements were assessed by Chi^2^, t-tests, or logistic regression analysis. Subsequently, a logistic regression model was used to evaluate the association of each variable with use of CAM and natural medicines/dietary supplements adjusted for the influence of other variables; the fully adjusted analysis included age, gender, educational level and household income. Results are presented as adjusted odds ratios (OR). Analysis was conducted using a significance level of 5%.

## Results

### Demographic details of study participants

Of the individuals identified in the Danish National Patient Registry and invited to participate in the pragmatic trial (n = 783), 247 took part in the trial (31.5% of invited individuals; men: n = 115, 46.6%; women: n = 132, 53.4%). Participants’ mean age was 64 (age range 36–80), with near equal distribution of those aged 64 and under, and those aged 65 and over; more women (n = 76; 30.8%) were in the younger age group, and more men (n = 71; 28.7%) in the older age group (X^2^(df 1) = 9.178; p = 0.002). The majority of participants (n = 148; 59.9%) had received chemotherapy and/or radiotherapy in addition to surgery (see Table 2).

**Table 2:** **Participants’ basic demographic details (n = 247)**

	Number of participants	%
**Sex:**		
Women	132	53.4
Men	115	46.6
**Age:**	Range [36–80]	
	Mean 64.06; SD [8.842]	
**Cancer treatment:**	Range [0–116; number of chemo/radio treatments]	
	Mean 14.02; SD [21.857]	
Surgery	99	40.1
Surgery + chemo/radio	148	59.9
**Months since Surgery:**		
0-5 months	54	21.9
6-9 months	61	24.7
10-13 months	68	27.5
14-17 months	64	25.9

### Attitudes to CAM and the use of alternative treatments and natural medicines/dietary supplements

Almost half of the participants in the trial as a whole (n = 247) considered themselves to have ‘very positive’ and ‘positive’ attitudes (45.5%) to CAM; 42% described their attitudes as ‘neutral’ and 12.4% as ‘negative’. In this study, 49.4% of respondents (n = 122; women: n = 79, 64.8%; men: n = 43, 35.2%) used some form of CAM in the past month. A quarter of respondents (25.1%; n = 62; women: n = 47; men: n = 15) had received some form of alternative treatment, while 40.1% of respondents (n = 99; women: n = 64; men: n = 35) used natural medicines and/or dietary supplements. Of those using CAM, 49.2% (n = 60) used only natural medicines/dietary supplements, 32% (n = 39) consulted an alternative therapist for treatment, and 18.9% (n = 23) used both.

Amongst the CAM users, more women than men received alternative treatments (women: n = 47; 75.8%; men: n = 15; 24.2%), with 66.1% aged 65 and under. As can be derived from Table 3, there was a statistically significant relationship between different levels and length of education: people with higher education (tertiary degree or higher) more frequently took recourse to alternative treatments than those with primary and lower secondary education only (77%, n = 47; 23%, n = 14; respectively, 1 participant missing).

**Table 3:** **Sociodemographic variables and educational level dichotomized (n = 122)**

	Alternative treatments						Natural medicines/dietary supplements					
	Unadjusted			Fully Adjusted^a^			Unadjusted			Fully Adjusted^a^		
	OR	P	CI	OR	P	CI	OR	P	CI	OR	P	CI
**Age**	**0.94**	.001	0.91-0.98	0.96	.064	0.94-1.00	0.98	.141	0.95-1.01	0.99	.743	0.96-1.03
**Gender**												
Male	Referent			Referent			Referent			Referent		
Female	**3.69**	<.001	1.93-7.06	**3.36**	.002	1.54-7.33	**2.15**	.004	1.27-3.63	**1.83**	.047	1.01-3.30
**Educational level**												
**< tertiary degree**	Referent			Referent			Referent			Referent		
**> tertiary degree**	**4.58**	<.001	2.35-8.90	**2.77**	.010	1.28-6.01	**2.23**	.003	1.32-3.78	1.59	.131	0.87-2.89
**Household income**	**1.33**	.001	1.13-1.57	1.13	.208	0.93-1.38	1.13	.110	0.97-1.30	1.05	.551	0.89-1.25

OR = Odds Ratios. CI = Confidence Interval. OR in bold differs significantly (95% CI) from the reference group (OR = 1.00).

^a^Fully adjusted analysis included age, gender, educational level, and household income in the multiple binary logistic regression analysis.

The majority (n = 64, 64.6%) of users of natural medicines and/or dietary supplements were also women (men: n = 35, 35.4%), with 57% aged 65 and under. As indicated in Table 3, a relationship between length and levels of education and the use of natural medicines/dietary supplements was noticeable, with 61 participants (62.9%) having had a tertiary education and 36 participants (37.1%) having had a lower or upper secondary education. That is to say, those who consulted alternative therapists were most commonly women (OR: 3.36; p = .002; CI: 1.54-7.33) with high educational levels (OR: 2.77; p = 0.010; CI: 1.28-6.01), and more women than men used natural medicines and/or dietary supplements (OR: 1.83; p = .047; CI: 1.01-3.30) independent of educational levels.

Amongst those consulting an alternative practitioner for treatment, the most common therapies were: massage (n = 26; 41.9%), acupuncture (n = 19; 30.6%), mindfulness/meditation (n = 17; 27.4%), reflexology (n = 9; 14.5%) and cranio-sacral therapy (n = 7; 11.3%). One or a combination of two therapies was used most frequently (58.1%, n = 36; 29%, n = 18; respectively); 12.8% (n = 8) used between 3 and 5 therapies. Respondents using natural medicines and/or dietary supplements commonly used fish oils (n = 43, 43%), vitamins (especially Vitamin C and D, and multi-vitamins) (n = 39, 39%), fibre (n = 20, 20%), and calcium (n = 16, 16%). Half of these respondents used one kind of natural medicines and/or dietary supplements and the other half combined two or three kinds, with vitamins, fish oils and calcium being popular combinations.

### Pathways to CAM use

Of the respondents receiving alternative treatments 66.1% (n = 41) self-initiated such treatments, whereas amongst those using natural medicines/dietary supplements 54% (n = 54) did so. Of lesser importance were suggestions by family and friends (alternative treatments 17.7% (n = 11); natural medicines/dietary supplements 22.2% (n = 22). Health professionals also suggested CAM use; 34.3% (n = 34) of respondents reported suggestions from health professionals on the use of natural medicines/dietary supplements, while 9.7% (n = 6) reported suggestions on the use of alternative treatments. Other sources of advice mentioned by respondents included CAM practitioners, other patients, patient organisations, work colleagues, and print and broadcast media.

### Reasons for the use or non-use of CAM

Amongst those respondents who used CAM (n = 122), the most frequent reason for CAM use concerned the aim to achieve ‘better physical well-being’ (51 responses; 41.8%). Also prominent was the wish ‘to do something actively’ (29 responses; 23.8%) and the consideration that ‘maybe CAM helps and doesn’t do any harm’ (29 responses; 23.8%). For further details, see Table 4.

**Table 4:** **Reasons for CAM use (n = 122)**

Reasons	Number of responses (n = 217; several responses possible)	%
Better physical well-being	51	41.8
Maybe it helps, and doesn’t do any harm	29	23.8
To do something actively	29	23.8
Support the body’s ability to fight the disease	24	18.9
Better mental/psychological well-being, e.g. to give hope, optimism	20	16.4
Counteract the negative side-effects of cancer or the medical treatment	17	13.9
To do everything to fight the disease	15	12.3
Effect on the disease itself	11	9
Other	21	15.6

Amongst those respondents who did not use CAM (n = 125) the most frequent reasons related to never having considered CAM use (83 responses; 66.4%) and being satisfied with conventional treatment (52 responses; 41.6%). For further details, see Table 5.

**Table 5:** **Reasons for non-use of CAM (n = 125)**

Reason	Number of responses (n = 177; several responses possible)	%
I never thought about having CAM	83	66.4
I am satisfied with the conventional treatment I received	52	41.6
I cannot afford to pay for CAM	13	10.4
I don’t believe in CAM, as it has not been tested thoroughly	12	9.6
I am having a break from using CAM	6	4.8
I stopped using CAM	2	1.6
I was advised against having CAM	1	0.8
Other	8	6.4

### Perceived effects of CAM

A majority of respondents experienced some beneficial effects that they attributed to CAM use (see Table 6); ‘no beneficial effects’ were reported only 13 times (10.7%). Beneficial effects were reported particularly in relation to perceived ‘better physical wellbeing’ (38 responses, 31.1%) and ‘the improvement of mental/psychological wellbeing’ (30 responses, 24.6%).

**Table 6:** **Perceived effects of CAM (n = 122)**

Perceived effects	Number of responses (n = 120; several responses possible)	%
Better physical well-being	38	31.1
An improvement of mental/psychological well-being, more hope and optimism	30	24.6
No beneficial effect	13	10.7
Reduction of side-effects from cancer and/or cancer treatment	10	8.2
Strengthening of body’s own ability to fight cancer	7	5.7
No direct effect on cancer	2	1.6
Other	20	16.4

Asked about harmful effects as a result of CAM use, a majority of respondents reported not to have experienced any negative consequences (83 responses; 68%). Negative effects were reported 9 times (7.3%) including ‘nausea and uneasiness’ , ‘gastric ulcer’ , ‘influence on blood samples’ , ‘weight loss’ , and ‘others’ (each reported in 1–3 responses); 24.7% respondents did not complete the question about perceived harmful effects.

### Communication about CAM with a medical doctor

Of those respondents using some form of CAM, 51.5% (n = 51) reported not to have informed their doctor about their CAM use. Of the whole study sample (n = 247) 8.5% (n = 21) reported to have been asked about CAM use by their doctor.

## Discussion

This is the first Danish study that examined the use of CAM by individuals who completed hospital treatment for colorectal cancer, and thus contributes to the under-researched field of CAM use for colorectal cancer. To contextualise the identified prevalence of CAM use among persons who had colorectal cancer, in the following we compare our findings with 1) studies of CAM use by Danish cancer patients in general, 2) the use of CAM in relation to breast and prostate cancer as examples of gender-specific cancers, and 3) international literature on CAM use among persons who had (or have) colorectal cancer.

Our study revealed a relatively high rate of CAM use after the completion of hospital treatment for colorectal cancer, when compared with the use of CAM by cancer patients generally in Denmark, and the use of CAM by Danish women diagnosed with breast cancer. In our study, 49.4% of participants used some form of CAM in the past month, a rate which is significantly higher than the use of CAM by cancer patients in general, reported to be 27% [8]; in turn, prevalence of CAM use by cancer patients across diverse cancers is only slightly higher than the use of CAM in the past year by the Danish population in general which was reported to be 26.3% (31.3% women; 21.1% men) in 2010 [22].

Our study identified that 64.8% women used some form of CAM in the past month. By comparison, a study of Danish women diagnosed with breast cancer reports that 40.1% of participating women used one or more types of CAM 3–4 months post-surgery [24]. Thus, women’s high rate of CAM use following colorectal cancer treatment when compared with the use of CAM by Danish breast cancer patients post-surgery seems surprising.

Women are well known, nationally and internationally, to be the main users of CAM [7, 22], including in cancer in Europe [21]. This raises the question of how our findings of high CAM use in colorectal cancer in Denmark compare with the prevalence of CAM use in the case of prostate cancer, the most common cancer to affect men [1], and where low CAM use could be anticipated. No Danish studies of CAM use in prostate cancer were identified, however a German study [25] reports that 47% of participants used some form of CAM within the past 4 weeks.^a^ These findings indicate that men’s use of CAM in prostate cancer in Germany is higher than men’s CAM use in colorectal cancer in Denmark (35.2%).

Lastly, when comparing our findings with international studies on the use of CAM in colorectal cancer the following can be noted: In different European countries, between 25% and 80% (mean 32%) of colorectal cancer patients (no gender details provided) are reported to use CAM post-diagnosis, with a sharp increase of CAM use noted following diagnosis [10]. Further, a population-based study in Alberta (Canada) reports that 69% of participants (59% women; 42% men) used some form of CAM after conventional care for colorectal cancer [13]. Neither of these studies confirms our findings, but diverse research designs and study populations, different national contexts, and heterogeneous notions of CAM limit international comparisons [7].

Given these diverse national, international and gender-specific findings and inconclusive comparisons, how can the results concerning the prevalence of CAM use in colorectal cancer in Denmark be interpreted? In the following, we suggest that a consideration of a study’s wider context reveals aspects that may explain participants’ high CAM use. Our study formed part of a pragmatic trial on energy healing as rehabilitation for colorectal cancer, whereby 45.5% of trial participants described their attitude to CAM as ‘very positive’ or ‘positive’ , and 42% as ‘neutral’. These attitudes to CAM may possibly indicate receptiveness to energy healing as a form of cancer rehabilitation, which – in turn - may also be reflected in the reported high use of CAM by male and female participants. Further, in Germany CAM is frequently practiced by medical doctors, enhancing the status of CAM and possibly implying positive attitudes to CAM [26]. This may lead to high rates of CAM use by German men with prostate cancer. Similarly, Tough el al ([13], p54) note that ‘having therapy options other than conventional treatment recommended by conventional doctors’ as one of the strongest predictors of CAM use in their sample. Therefore, akin to our study and Bruns et al’s [25] sample, Tough et al’s [13] sample seems to be characterized by particular experiences and attitudes towards CAM which may be implicated in participants’ high prevalence of CAM use after conventional care for colorectal cancer. In summary, the relatively high use of CAM in our study, as well as in Tough et al’s [13] and Bruns et al’s studies [25], may be the result of ‘facilitative’ circumstances, that is, study participants’ and/or their medical professionals’ positive attitudes to CAM (see also [26]).

Beyond prevalence of CAM use, our study also identified other patterns related to CAM use following treatment for colorectal cancer. The majority of respondents self-initiated their use of CAM or followed suggestions by family and friends. The importance of social networks in prompting CAM use is well established in the wider literature on CAM use [26], including for cancer patients in Denmark [8]. In particular, in countries where CAM is largely practiced outside national healthcare provision, such as Denmark, citizens tend to use their social network as main information source, although they may also wish to receive information about CAM from biomedical professionals [26].

In using CAM, the respondents in our study, like other cancer patients in Denmark [8, 9], aimed to address physical concerns and symptoms and to contribute actively to improving their health and well-being. The wish to be actively involved in caring for one’s health is frequently seen by CAM users generally as one of the attractions of CAM [26]. On the other hand, ‘never having thought about using CAM’ was given by respondents in our study as the most common reason for not using CAM. The lack of information about CAM and/or specific CAM therapies is also recognized in other studies as centrally contributing to the non-use of CAM [26]. As a result, some cancer patients express a wish for more, and particularly trustworthy, information, for example through telephone advice lines [8] or through national health service provision [27]. In the absence of such information, CAM users predominantly rely on personal experiences of, for instance, the perception of beneficial (and/or harmful) effects of CAM, as reported here and also previously by others [8, 9, 26].

About half of the respondents in our study, like other cancer patients in Denmark [8, 9], did not disclose their CAM use to their physician. This is not to suggest that users would not like to discuss their CAM use with their doctor or oncologist [8, 9, 26], but may reflect users’ concerns about or experiences of negative reactions to their choice of healthcare [9, 15, 26] and some biomedical professionals’ lack of knowledge about CAM (for details, see [28]). The literature on patterns of disclosure of CAM use to biomedical professionals also indicates CAM users’ understanding that CAM addresses other issues and problems than biomedical treatment, or that biomedical providers do not need to know about CAM use [26]. This suggests that more accessible and reliable information about CAM and improved communication in the clinical context about the use of CAM and acknowledgment of its widespread use in colorectal cancer is of relevance to patients and professionals alike. EU patients have the right to receive comprehensive and easily understood information about their health and healthcare from their healthcare provider [29] and patients’ access to diverse, reliable and trustworthy information, including about CAM, increases their ability to make informed healthcare decisions [26, 28]. At the same time, biomedical providers’ increased knowledge about CAM and the obtaining of details about patients’ CAM use may prevent drug interactions and potentially adverse interactions with anti-cancer treatments [11, 12].

### Limitations

Some limitations to the questionnaire study presented can be noted. A selection bias may have influenced the results presented, as only 31.5% of eligible individuals from a national Danish cohort participated (for details on trial participants and non-participants, see [30]). Further, since participants had completed hospital treatment for colorectal cancer up to 18 months prior to study inclusion it is difficult to ascertain whether their CAM use within the past month related to the cancer from which they had suffered. In addition, in Denmark, a distinction is made between alternative treatments, natural medicines and dietary supplements at the juridical level [23, 31]; respondents may however have used these terms interchangeably, leading to an over- or underreporting of different forms of CAM use. Also, those respondents using particular natural medicines and/or dietary supplements may not think of these products as medicines or a supplement, possibly underreporting their use.

## Conclusion

The use of CAM following completion of hospital treatment for colorectal cancer seems widespread in Denmark. Of 247 individuals who completed hospital treatment for colorectal cancer in Denmark, 49.4% (64.8% women; 35.2% men) used some form of CAM within the past month; of those using CAM, 49.2% used only natural medicines/dietary supplements, 32% consulted an alternative therapist for treatments, and 18.9% used both. Women with higher educational levels were the typical users of alternative treatments provided by an alternative practitioner; more women than men used natural medicines and/or dietary supplements independent of educational levels. The identified widespread use of CAM suggests a need for more reliable and diverse information about CAM and improved communication in the clinical context. Addressing these issues can support patients’ rights to healthcare information and their wish to make informed healthcare decisions, as well as biomedical professionals’ endeavours to protect patients from potentially adverse interactions between biomedical treatment and CAM treatments or natural medicines and/or dietary supplements.

## Endnote

^a^To enhance comparability across the different studies discussed here, studies selected for comparison were restricted to those that most closely resemble our study design and geographical region. For other studies, see [32].

## Electronic supplementary material

Additional file 1: **Questionnaire: Alternativ behandling.** (PDF 117 KB)
