# Supplementary material for: The use of complementary and alternative medicine after the completion of hospital treatment for colorectal cancer: findings from a questionnaire study in Denmark
Source: BMC Complement Altern Med. 2014 Oct 10;14:388. doi: 10.1186/1472-6882-14-388 (PMC4197266; doi:10.1186/1472-6882-14-388)
Supplement: Supplementary file 1 — Additional file 1: Questionnaire: Alternativ behandling. (PDF 117 KB) [file 12906_2014_1954_MOESM1_ESM.pdf]

5302297097

ALTERNATIV BEHANDLING

B

1. Har du inden for <u>den sidste måned</u> brugt nogen form for alternativ og/eller komplementær behandling?

	Ja	Nej	Ved ikke
a) Massage, osteopati eller andre manipulative terapier.........	☐	☐	☐
b) Zoneterapi........	☐	☐	☐
c) Akupunktur........	☐	☐	☐
d) Healing, håndspålæggelse og lignende........	☐	☐	☐
e) Kraniosakral terapi........	☐	☐	☐
f) Homeøpati........	☐	☐	☐
g) Ernæringsterapi/diætbehandling........	☐	☐	☐
h) Kinesiologi........	☐	☐	☐
i) Hypnose........	☐	☐	☐
j) Mindfulness/meditation........	☐	☐	☐
k) Andet: ______________________	☐		

<u>Hvis JA</u>, på hvilket initiativ anvender du ovenstående behandling *(sæt gerne flere krydser)*:

- ☐ Jeg anvender alternativ og/eller komplementær behandling på eget initiativ
- ☐ Anbefalet af læge/sygeplejerske
- ☐ Anbefalet af alternativ behandler
- ☐ Anbefalet af andre patienter
- ☐ Anbefalet af familie og/eller venner
- ☐ Anbefalet ved kontakt til patientforening/sammenslutning
- ☐ Andet: ______________________

2. Har du inden for <u>den sidste måned</u> anvendt naturlægemidler og/eller kosttilskud (dvs. piller, kapsler, medicinsk te, ekstrakter og lignende)**?**

☐ Ja ☐ Nej ☐ Ved ikke

- sp. 2 fortsættes næste side

ALTERNATIV BEHANDLING

- sp. 2 fortsat

Hvis JA, anfør venligst i boksen herunder de specifikke typer af naturlægemidler og kosttilskud, som du bruger:

Hvis JA, på hvilket initiativ tager du naturlægemidler og/eller kosttilskud *(sæt gerne flere krydser)*:

- ☐ Anbefalet af alternative behandler
- ☐ Jeg har købt det på eget initiativ
- ☐ Anbefalet af læge/sygeplejersker
- ☐ Anbefalet af andre patienter
- ☐ Anbefalet af familie og/eller venner
- ☐ Anbefalet ved kontakt til patientforening/sammenslutning
- ☐ Andet: ______________________

HVIS DU HAR KRYDSET NEJ HELE VEJEN NED I SPØRGSMÅL 1 OG I SPØRGSMÅL 2, GÅ DA TIL SPØRGSMÅL 7

3. Har du fortalt din behandlende læge, at du bruger alternativ behandling?

☐ Ja ☐ Nej

4. Angiv venligst grundene til at du bruger alternativ behandling *(sæt alle de krydser der passer for dig)*:

☐ For at opnå effekt på selve sygdommen	☐ For et bedre psykisk velbefindende, give håb optimisme osv.	☐ For at gøre alt hvad der er muligt for at bekæmpe sygdommen
☐ For at øge kroppens evne til at bekæmpe sygdommen	☐ For at modarbejde dårlige virkninger fra kræften eller den medicinske behandling	☐ For selv at gøre noget aktivt
☐ For et bedre fysisk velbefindende	☐ Fordi det måske kan hjælpe og ikke skade	☐ Andet (uddyb nedenfor)

Hvis kryds i Andet, uddyb: ______________________

ALTERNATIV BEHANDLING

5. Hvilke <u>gavnlige virkninger</u> har du faktisk oplevet ved den alternative behandling?
(sæt alle de krydser der passer for dig):

- ☐ Ingen gavnlig virkning
- ☐ En direkte effekt på kræftsygdommen (eks. mindskning af kræfttumoren)
- ☐ Et bedre fysisk velbefindende
- ☐ En bedring af psykisk velbefindende, håb, optimisme med mere
- ☐ Reduktion af bivirkninger fra kræftsygdommen og/eller den medicinske behandling
- ☐ Kroppens egen evne til at bekæmpe sygdommen er øget
- ☐ Andet ______________________________

6. Hvilke <u>skadelige virkninger</u> har du oplevet ved den alternative behandling?
(sæt alle de krydser der passer for dig):

- ☐ Ingen skadelig virkning
- ☐ Kvalme og utilpashed
- ☐ Vægttab
- ☐ Påvirkning af blodprøver
- ☐ Mavesår
- ☐ Andet: ______________________________

7. Hvis du i <u>spørgsmål 1 og spørgsmål 2 svarede NEJ</u>, hvad er da hoved-årsagen til, at du for øjeblikket ikke bruger alternativ behandling?
(sæt ét kryds)

- ☐ Har aldrig tænkt på at bruge alternativ behandling
- ☐ Jeg tror ikke på behandlinger, som ikke er gennemafprøvede
- ☐ Jeg er tilfreds med udsigterne for den etablerede behandling jeg får/har fået
- ☐ Jeg har ikke råd til at betale for alternativ behandling
- ☐ Jeg har tænkt på at bruge alternativ behandling, men er blevet frarådet af familie/venner/personale i sundheds-sektoren, og besluttede derfor ikke at gøre det
- ☐ Jeg har tidligere i mit sygdomsforløb brugt alternativ behandling, men holder nu en pause
- ☐ Jeg har tidligere i mit sygdomsforløb brugt alternativ behandling, men er nu stoppet
- ☐ ANDET:

8. Har din behandlende læge spurgt dig, om du anvender alternativ behandling?

☐ Ja ☐ Nej
